# Association of polymorphisms in the telomere-related gene ACYP2 with lung cancer risk in the Chinese Han population

**DOI:** 10.18632/oncotarget.13870

**Published:** 2016-12-10

**Authors:** Nanzheng Chen, Xiaomei Yang, Wen Guo, Jiangtao You, Qifei Wu, Guangjian Zhang, Haijun Li, Donghong Geng, Tianbo Jin, Junke Fu, Yong Zhang

**Affiliations:** ^1^ Department of Thoracic Surgery, The First Affiliated Hospital of Xi’an Jiaotong University, Xi’an 710061, Shaanxi, China; ^2^ Hospital 521 of China's Ordnance Industry Group, Xi’an 710065, Shaanxi, China; ^3^ Inner Mongolia Medical University, Hohhot 010050, Inner Mongolia, China; ^4^ School of Continuing Education of Xi’an Jiaotong University, Xi’an 710061, Shaanxi, China; ^5^ School of Life Sciences, Northwest University, Xi’an 710069, Shaanxi, China

**Keywords:** association study, lung cancer, telomere-related gene, single nucleotide polymorphisms (SNPs)

## Abstract

Single nucleotide polymorphisms (SNPs) in the telomere-associated gene *ACYP2* are associated with increased lung cancer risk. We explored the correlation between *ACYP2* SNPs and lung cancer susceptibility in the Chinese Han population. A total of 554 lung cancer patients and 603 healthy controls were included in this study. Thirteen SNPs in *ACYP2* were selected. Odds ratios (ORs) and 95% confidence intervals (95% CIs) were calculated using unconditional logistic regression analysis. Multivariate logistic regression analysis was used assess the correlation between SNPs and lung cancer. We found that rs1682111 was associated with increased lung cancer risk in the recessive model (crude, OR=1.50, 95%CI: 1.04-2.16, p=0.029; adjusted for age, OR=1.55, 95%CI: 1.04-2.30, p=0.029), as was rs11896604 in the codominant model (crude, OR=0.65, 95%CI: 0.33-1.28, p=0.045; adjusted for age, OR=0.74, 95%CI: 0.36-1.53, p=0.049) and over-dominant model (crude, OR=1.30, 95%CI: 1.02-1.66, p=0.032; adjusted for age, OR=1.37, 95%CI: 1.05-1.78, p=0.020). Finally, rs843720 was associated with increased lung cancer risk in the recessive model (crude, OR=1.43, 95%CI: 1.02-2.02, p=0.040; adjusted for age, OR=1.48, 95%CI: 1.02-2.15, p=0.040). Thus three SNPs in *ACYP2* (rs1682111, rs11896604 and rs843720) associate with lung cancer in the Chinese Han population.

## INTRODUCTION

Lung cancer, the leading cause of cancer-related death in most countries, has been increasing rapidly in China for the last three decades [1]. The risk of lung cancer is strongly associated with exposure to environmental carcinogens, in particular to cigarette smoke [2]. However, only approximately 10% of smokers ever develop lung cancer. This highlights the potential role of hereditary components in determining the risk of lung cancer [3]. Through genome-wide association studies (GWAS), a number of genes involved in lung carcinogenesis have been identified in populations of European descent. These include *CHRNA5*, *CHRNA3* and *CHRNA4* on chromosome 15q25.1 [4–6], (*TERT-CLPTM1L*) on 5p15.33 [7, 8], and (*BAT3-MSH5*) on 6p21.33 [7].

It was also learned from GWAS that inherited single nucleotide polymorphisms (SNPs) in telomere-related genes are associated with various malignancies [9]. Human telomeres, composed of a tandem hexanucleotide repeat (TTAGGG), are many kilobases long in newborns but shorten by an average of 20-40 base-pairs annually [10]. SNPs in the telomere-related gene *ACYP2* are reproducibly associated with inter-individual variation in leukocyte telomere length (LTL) [11]. In addition, recent Mendelian randomization studies indicate that LTL-associated SNPs related to longer telomere lengths are associated with increased risk of adult glioma, melanoma and lung cancer [12].

As far as we know, no studies have investigated the correlations between SNPs in *ACYP2* and lung cancer susceptibility. We therefore performed a case-control association study to determine whether any of 13 SNPs in the *ACYP2* gene are associated with lung cancer susceptibility in the Chinese Han population.

## RESULTS

### Participant characteristics

A total of 554 lung cancer patients (416 males, 138 females; median age at diagnosis 48.24 ± 13.05 years) and 603 healthy subjects (469 males, 134 females; median age 58.18 ± 10.53 years) were enrolled in our study. There is significant difference in age between patients in the case and control groups (*p*<0.001), but no difference in gender (Table 1).

**Table 1:** Characteristics of the cases and controls

Variables	Case (N=554)	Control (N=603)	Total	*p*
Gender, No. (%)				0.282^a^
Male	416 (75.1%)	469 (77.8%)	885	
Female	138 (24.9%)	134 (22.2%)	272	
Mean age ±SD	48.24 ± 13.05	58.18 ± 10.53		<0.001^b^

^a^ *p* was calculated using the chi-square test.

*^b^ p* was calculated using Student's t-test.

### Association between *ACYP2* polymorphisms and lung cancer risk

The minor allele of each SNP was assumed to be a risk factor. The minor allele frequencies (MAF) are listed in Table 2. As a risk factor, the minor allele of each SNP was compared with the wild-type allele. All of the tested SNPs were consistent with the Hardy-Weinberg equilibrium for the control population of this study (*p*>0.05). Comparing the differences in the frequency distributions of the alleles between the cases and controls using the χ^2^ test, we found no correlation between the loci and lung cancer susceptibility.

**Table 2:** Basic information on the candidate SNPs

Rs number	Nucleotide position	Allele A/B	MAF frequency		HW *p*	Allele model		*p*
			case	control		OR	95%CI	
rs6713088	54345469	G/C	0.404	0.393	0.089	1.048	0.887-1.237	0.585
rs12621038	54391113	T/C	0.454	0.465	0.368	0.956	0.812-1.126	0.591
rs1682111	54427979	A/T	0.347	0.309	0.924	1.189	1.000-1.415	0.051
rs843752	54446587	G/T	0.234	0.260	0.089	0.870	0.720-1.052	0.151
rs10439478	54459450	C/A	0.430	0.433	0.245	0.987	0.837-1.164	0.880
rs843645	54474664	G/T	0.225	0.250	0.065	0.874	0.721-1.059	0.169
rs11125529	54475866	A/C	0.195	0.187	0.503	1.054	0.857-1.298	0.617
rs12615793	54475914	A/G	0.211	0.200	0.702	1.072	0.876-1.312	0.499
rs843711	54479117	T/C	0.436	0.448	0.510	0.953	0.809-1.123	0.567
rs11896604	54479199	G/C	0.214	0.200	0.800	1.087	0.889-1.330	0.415
rs843706	54480369	A/C	0.432	0.452	0.459	0.925	0.784-1.091	0.355
rs17045754	54496757	C/G	0.196	0.187	1.000	1.062	0.863-1.307	0.570
rs843720	54510660	G/T	0.380	0.343	0.527	1.171	0.988-1.388	0.068

SNPs: Single nucleotide polymorphisms; MAF: Minor allele frequency; HWE: Hardy-Weinberg equilibrium; OR: Odds ratio; CI: Confidence interval; A: Minor alleles; B: Major alleles.

After adjusting for age, the models were further analyzed using unconditional logistic regression analysis of SNPs associated with lung cancer (Table 3). We found that the AA genotype of rs1682111 was associated with increased lung cancer risk under the recessive model (crude, OR=1.50, 95%CI: 1.04-2.16, *p*=0.029; adjusted for age, OR=1.55, 95%CI: 1.04-2.30, *p*=0.029). We also noticed that rs11896604 was associated with lung cancer risk in codominant model (“GG”, crude, OR=0.65, 95%CI: 0.33-1.28, *p*=0.045; adjusted for age, OR=0.74, 95%CI: 0.36-1.53, *p*=0.049) and over-dominant model (“CG”, crude, OR=1.30, 95%CI: 1.02-1.66, *p*=0.032; adjusted for age, OR=1.37, 95%CI: 1.05-1.78, *p*=0.020). In addition, the GG genotype of rs843720 increased the risk of lung cancer under the recessive model (crude, OR=1.43, 95%CI: 1.02-2.02, *p*=0.040; adjusted for age, OR=1.48, 95%CI: 1.02-2.15, p=0.040).

**Table 3:** Single loci associated with lung cancer (adjusted by age)

snps	Model	Genotype	Controls(n%)	Cases(n%)	Without adjustment		With adjustment	
					OR (95% CI)	p1	OR (95% CI)	p2
rs1682111	Codominant	T/T	287 (47.6%)	244 (44.0%)	1[Ref]		1[Ref]	
		T/A	259 (43%)	235 (42.4%)	1.07 (0.83-1.36)		1.01 (0.78-1.32)	
		A/A	57 (9.4%)	75 (13.5%)	1.55 (1.05-2.27)	0.080	1.56 (1.03-2.37)	0.092
	Dominant	T/T	287 (47.6%)	244 (44.0%)	1[Ref]		1[Ref]	
		T/A-A/A	316 (52.4%)	310 (56.0%)	1.15 (0.9-21.45)	0.230	1.11 (0.86-1.43)	0.420
	Recessive	T/T-T/A	546 (90.5%)	479 (86.5%)	1[Ref]		1[Ref]	
		A/A	57 (9.4%)	75 (13.5%)	1.50 (1.04-2.16)	0.029	1.55 (1.04-2.30)	0.029
	Over-dominant	T/T-A/A	344 (57%)	319 (57.6%)	1[Ref]		1[Ref]	
		T/A	259 (43%)	235 (42.4%)	0.98 (0.77-1.24)	0.850	0.93 (0.72-1.19)	0.550
	Log-additive	—	—	—	1.18 (1.00-1.40)	0.054	1.17 (0.97-1.41)	0.100
rs11896604	Codominant	C/C	386 (64.1%)	331 (59.8%)	1[Ref]		1[Ref]	
		C/G	191 (31.7%)	209 (37.7%)	1.28 (1.00-1.63)		1.35 (1.03-1.76)	
		G/G	25 (4.2%)	14 (2.50%)	0.65 (0.33-1.28)	0.045	0.74 (0.36-1.53)	0.049
	Dominant	C/C	386 (64.1%)	331 (59.8%)	1[Ref]		1[Ref]	
		C/G-G/G	216 (35.9%)	223 (40.2%)	1.20 (0.95-1.53)	0.130	1.28 (0.99-1.66)	0.062
	Recessive	C/C-C/G	577 (95.8%)	540 (97.5%)	1[Ref]		1[Ref]	
		G/G	25 (4.2%)	14 (2.50%)	0.60 (0.31-1.16)	0.120	0.66 (0.32-1.37)	0.260
	Over							
	dominant	C/C-G/G	411 (68.3%)	345 (62.3%)	1[Ref]		1[Ref]	
		C/G	191 (31.7%)	209 (37.7%)	1.30 (1.02-1.66)	0.032	1.37 (1.05-1.78)	0.020
	Log-additive	—	—	—	1.09 (0.89-1.34)	0.400	1.16 (0.92-1.45)	0.200
rs843720	Codominant	T/T	256 (42.5%)	217 (39.2%)	1[Ref]		1[Ref]	
		G/T	280 (46.4%)	252 (45.6%)	1.06 (0.83-1.36)		1.07 (0.82-1.41)	
		G/G	67 (11.1%)	84 (15.2%)	1.48 (1.02-2.14)	0.110	1.54 (1.03-2.29)	0.110
	Dominant	T/T	256 (42.5%)	217 (39.2%)	1[Ref]		1[Ref]	
		G/T-G/G	347 (57.5%)	336 (60.8%)	1.14 (0.90-1.44)	0.270	1.16 (0.90-1.50)	0.250
	Recessive	T/T-G/T	536 (88.9%)	469 (84.8%)	1[Ref]		1[Ref]	
		G/G	67 (11.1%)	84 (15.2%)	1.43 (1.02-2.02)	0.040	1.48 (1.02-2.15)	0.040
	Over							
	dominant	T/T-G/G	323 (53.6%)	301 (54.4%)	1[Ref]		1[Ref]	
		G/T	280 (46.4%)	252 (45.6%)	0.97 (0.77-1.22)	0.770	0.97 (0.75-1.25)	0.810
	Log-additive	—	—	—	1.17 (0.99-1.39)	0.068	1.19 (0.99-1.43)	0.064

Finally, we also performed a Wald test using unconditional multivariate regression analysis to assess the associations between SNP haplotypes and lung cancer risk. However, no positive results were observed.

## DISCUSSION

In this study, three SNPs (rs1682111, rs11896604 and rs843720) were found to be associated with the lung cancer susceptibility in the Chinese Han population. Rs1682111 was associated with increased lung cancer risk in the recessive model. An association between this locus and other diseases has not been previously reported. Although this is the first report that rs11896604 associates with lung cancer susceptibility in the codominant over-dominant model, it was previously suggested that rs11896604 associates with a decreased risk of breast cancer [13]. In addition, rs843720 associated with an increased the risk of lung cancer in the recessive model.

*ACYP2*, located on chromosome 2p16.2, encodes a small cytosolic enzyme acylphosphatase that catalyzes the dephosphorylation of phospho-enzyme intermediates of various membrane pumps, particularly the Ca^2+^/Mg^2+^-ATPase from sarcoplasmic reticulum of skeletal muscle [14]. The physiological function of *ACYP2* is not yet completely clear, however. Vos et al. reported that the rs1872328 variant of *ACYP2* was associated with cisplatin-induced ototoxicity in patients with osteosarcoma who did not receive potentially ototoxic cranial irradiation [15]. It was further suggested that severe oxaliplatin-induced chronic peripheral neurotoxicity was potentially associated with *ACYP2* rs843748 [16]. A recent genome-wide meta-analysis showed that *ACYP2* rs11125529 affects telomere length and coronary heart disease in the Chinese Han population [17]. In addition, Yongjun et al found that *ACYP2* rs12615793 and rs11896604 may significantly decrease the risk of high-altitude pulmonary edema [18]. However, the mechanism by which *ACYP2* contributes to lung cancer will need to be explored in future studies.

Although the present study has several strengths, it also has intrinsic limitations. First, we present no data on the relation between *ACYP2* SNPs and the most influential lung cancer risk: smoking. Second, the size of our sample is relatively small. Finally, other factors such as BMI, bacterial and viral infection, marriage, and economic status were not taken into account. Those issues will be addressed in future studies, along with further characterization of the molecular mechanism underlying the association of *ACYP2* SNPs and lung cancer and prospective studies to validate their clinical utility.

In sum, we have identified several novel associations between three SNPs in *ACYP2* (rs1682111, rs11896604 and rs843720) and lung cancer in the Chinese Han population. Our findings suggest *ACYP2* may be a useful marker that informs clinical decisions, and may shed light on new candidate genes and new ideas about the mechanism governing the occurrence of lung cancer.

## MATERIALS AND METHODS

### Study participants

We recruited 554 patients with lung cancer and 603 healthy controls for this study. The patients were treated at the First Affiliated Hospital of Xi’an Jiao Tong University between January 2014 and August 2016. All demographic and related clinical data, including residential region, age, ethnicity, and education status, were collected through a face-to-face questionnaire and review of medical records. Patients recently diagnosed with primary lung cancer (confirmed by histopathological analysis) were included. Patients with other types of cancers or who underwent radiotherapy or chemotherapy were excluded. The controls underwent annual health evaluations in the checkup centers affiliated with our institution. All control patients were in good health and had no history of cancer, and they had no blood relatives with lung cancer going back three generations. This research was performed in accordance with the Chinese Department of Health and Human Services regulations for the protection of human research subjects. We obtained informed consent from all of the participants, and the study protocols were approved by the Institutional Review Board of Xi’an Jiao Tong University.

### SNP selection and genotyping

Thirteen SNPs in *ACYP2* that had a MAF >5% in the HapMap Asian population were selected for the association analysis [13, 14]. Venous blood samples (5 mL) were collected from each study participant during a laboratory examination. Blood samples from patients were collected prior to radiation or chemotherapy. DNA was extracted from whole blood samples using a Gold Mag-Mini Whole Blood Genomic DNA Purification Kit (GoldMag Ltd, Xian, China) and stored at –80°C after centrifugation. The DNA concentration was measured using spectrometry (DU530 UV/VIS spectrophotometer, Beckman Instruments, Fullerton, CA, USA). Sequenom MassARRAY Assay Design 3.0 software (Sequenom, Inc, San Diego, CA, USA) was used to design the multiplexed SNP Mass EXTEND assay, and genotyping was performed using a Sequenom MassARRAY RS1000 (Sequenom, Inc.) according to the manufacturer's protocol. SequenomTyper 4.0 Software™ (Sequenom, Inc.) was used to manage and analyze the data. The primers corresponding to each SNP are shown in Table 4. Based on these results, the following 13 SNPs were selected: rs6713088, rs12621038, rs1682111, rs843752, rs10439478, rs843645, rs11125529, rs12615793, rs843711, rs11896604, rs843706, rs17045754 and rs843720. Basic information on the SNPs is shown in Table 2.

**Table 4:** Primers used

SNP_ID	1st-PCRP	2nd-PCRP	UEP_SEQ
rs6713088	ACGTTGGATGACACACACAGACTCCTTCAC	ACGTTGGATGGTCACCAAAACACGTAATG	gaggcCAGAATGGTCCACTAGAGA
rs12621038	ACGTTGGATGATTGTGCTAGGCACTTTAGG	ACGTTGGATGGGCATAAGTTTTATTGCCTC	ccATTGCCTCAGCTAGACT
rs1682111	ACGTTGGATGGAATTGCTGGGTTATTTGGC	ACGTTGGATGGCCAGTGGGAATGCAAAATG	tgtcATGCAAAATGAAACAGACACTT
rs843752	ACGTTGGATGTCCTCTTTTCAGAAACCTGC	ACGTTGGATGGAGACAACATAATGGAGGTC	cGAGTTTGGGTTTGAGGT
rs10439478	ACGTTGGATGTAGCACAAGACCTACACTGG	ACGTTGGATGCTACACTCTCCAGAGGAATG	TTGCTGTTTTCCCAGAA
rs843645	ACGTTGGATGGAAATCTGAATACCACCTAC	ACGTTGGATGACAGTGCCTTTAGCAAGGTG	TCATAGGCACTACTGTATC
rs11125529	ACGTTGGATGCCGAAGAAAAGAAGATGAC	ACGTTGGATGGAGCTTAGTTGTTTACAGATG	AGAAAAGAAGATGACTAAAACAT
rs12615793	ACGTTGGATGATCTTGGCCCTTGAAGAA	ACGTTGGATGTTTGAGCTTAGTTGTTTAC	AAATTGAGTGACAAATATAAACTAC
rs843711	ACGTTGGATGGACAAAGGACCTTACAACTC	ACGTTGGATGTGCCTTGTGGGAATTAGAGC	gggaTCAGGGAACCAGTGCAAA
rs11896604	ACGTTGGATGAAGTCAGAATAGTGCTTAC	ACGTTGGATGTGTCTCTGACCTAGCATGTA	GTTAAGCTTGCAAGGAG
rs843706	ACGTTGGATGTGAAAGCCATAAATATTTTG	ACGTTGGATGTGAATAACTTGGTCTTATC	cACTTGGTCTTATCTGATGC
rs17045754	ACGTTGGATGCTGTAAAAGTTCTGGCATGG	ACGTTGGATGGAAATCAGGGATATTAGTGC	caggTATTCAGCTTCCTAGAGTTA
rs843720	ACGTTGGATGCTTCACAACACTCCTGTAAG	ACGTTGGATGAGTCAGAGCTAGACCTCTGG	ccccAATCTGTCTCAGGGTCTT

### Statistical analysis

We used Chi-squared tests (categorical variables) and Student's t-tests (continuous variables) to assess the differences in the demographic characteristics between the cases and controls. The Hardy-Weinberg equilibrium of each SNP was assessed in order to compare the expected frequencies of the genotypes in the control groups. All minor alleles were regarded as risk alleles for lung cancer susceptibility. To evaluate associations between each SNP and lung cancer risk in the five models (codominant, dominant, recessive, over-dominant and log-additive), ORs and 95% CIs were calculated using unconditional logistic regression analysis after adjusting for age, and gender. Linkage disequilibrium analysis and SNP haplotypes were analyzed using the Haploview software package (version 4.2) and the SHEsi software platform (http://www.nhgg.org/analysis/). All statistical analyses were performed using the SPSS version 17.0 statistical package (SPSS, Chicago, IL, USA) and Microsoft Excel. All statistical tests were two-sided, and values of *p*<0.05 was considered significant.
